# Abdominal hemorrhage and vascular fragility associated with neurofibromatosis type 1

**DOI:** 10.1007/s11604-025-01738-0

**Published:** 2025-01-24

**Authors:** Haruka Sato, Fumito Okada, Yoshiki Asayama

**Affiliations:** 1 Department of Radiology, Faculty of Medicine, Oita University, 1-1 Idaigaoka, Hasama-machi, Yufu, Oita 879-5593 Japan; 2 Department of Radiology, Oita Prefectural Hospital, Oita, Japan

**Keywords:** Neurofibromatosis type 1, NF-1 vasculopathy, Vascular fragility

## Abstract

We report a case of recurrent abdominal bleeding associated with vascular fragility in a 67-year-old woman with neurofibromatosis type 1 (NF-1). Computed tomography (CT) scan revealed hemorrhagic ascites and a pseudoaneurysm of the sigmoid colon artery, which was suspected to be the source of bleeding. Emergency laparotomy confirmed extremely fragile vessels, requiring repeated surgeries for recurrent bleeding. The patient was diagnosed with NF-1 vasculopathy, a rare vascular complication of this autosomal dominant disorder. NF-1, which affects 1 in 3000–5000 individuals, is associated with reduced life expectancy due to malignancies and vascular diseases, including NF-1 vasculopathy. This condition involves structural vascular abnormalities and increased fragility affecting vessels of all sizes. Recognition of this fragility is critical during invasive procedures, such as interventional radiology or surgery, to reduce the risk of bleeding and ensure optimal management. This case highlights the importance of considering NF-1 vasculopathy in patients with abdominal bleeding and adopting tailored strategies to address its challenges.

A 67-year-old woman with neurofibromatosis type 1 (NF-1) presented to the emergency department with abdominal pain. A computed tomography scan of the abdomen revealed massive bloody ascites and a pseudoaneurysm in a branch of the sigmoid colon artery (Fig. 1a and b), identified as the source of the bleeding. She had experienced a similar episode 2 years earlier, which was managed with coil embolization. This time, an emergency laparotomy was performed to resect the lesion. The lesion and the blood vessels in the operative area were particularly fragile, leading to repeated episodes of bleeding. Four hours after the first surgery, the following day, and 3 days later, the patient underwent additional laparotomies for management of further episodes of bleeding, indicating severe vascular fragility. She was diagnosed with NF-1 vasculopathy presenting as recurrent intra-abdominal bleeding and vascular fragility.

**Fig. 1:**
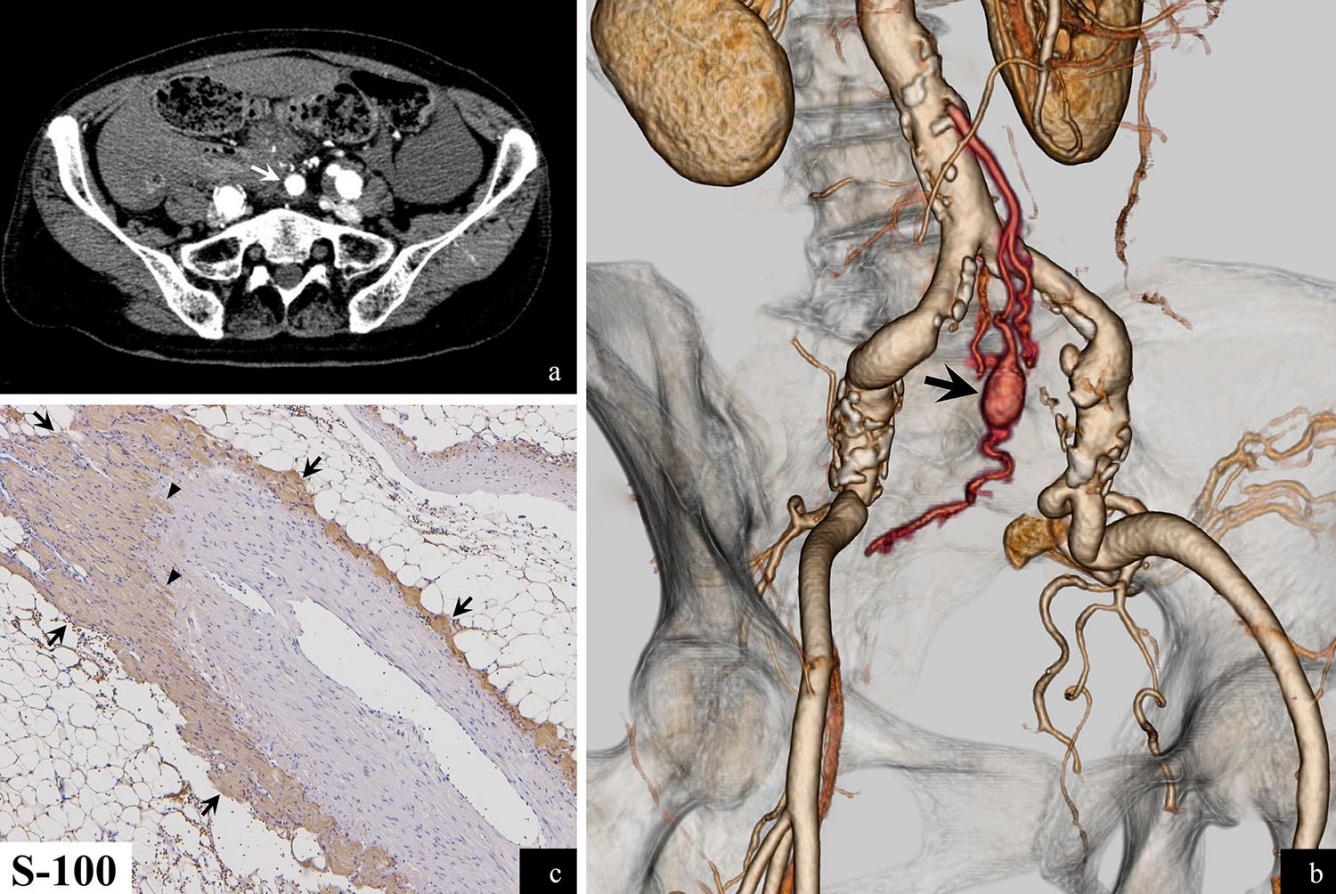
(**a**) (**b**) Contrast abdominal CT scan and volume rendering image of CT angiogram (**c**) S-100 stained histopathological image

NF-1 is a systemic autosomal dominant disorder characterized by neurofibromas (Fig. 2a and b) and café-au-lait spots [1]. Patients with NF-1 have shorter lifespans than the general population. The most common cause of death is malignancy, including malignant peripheral nerve sheath tumors and breast cancer (the patient had a history of breast cancer) (Fig. 2a), followed by vascular disease. This form of vascular disease, known as NF-1 vasculopathy, can involve both large and small arteries from the aorta to small branches; it may also affect veins [2]. Its pathogenesis remains unclear. However, in this case, histopathology revealed neurofibromas surrounding and invading the affected artery (Fig. 1c), suggesting vasculopathy due to direct vascular invasion.

**Fig. 2:**
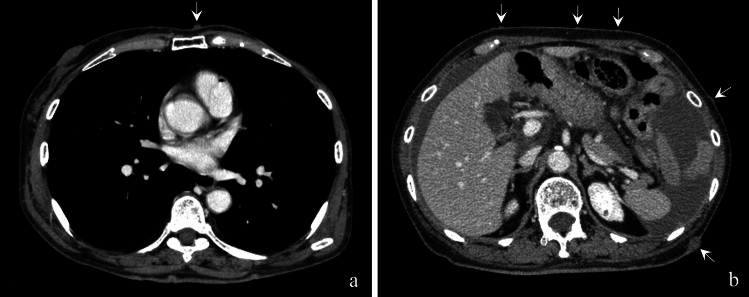
(**a**) Contrast chest CT scan (**b**) Contrast abdominal CT scan

NF-1 vasculopathy may lead to vascular fragility, an increased risk of bleeding during invasive procedures, and difficulty in achieving hemostasis. When performing invasive procedures, such as interventional radiology, it is essential to recognize the vascular fragility associated with NF-1 vasculopathy and to manage it accordingly.
